# Effect of pulmonary vein isolation on rotor/multiple wavelet dynamics in persistent atrial fibrillation, association with vagal response and implications for adjunctive ablation

**DOI:** 10.1007/s00380-022-02209-6

**Published:** 2022-11-27

**Authors:** Asuka Nishimura, Masahide Harada, Takashi Ashihara, Yoshihiro Nomura, Yuji Motoike, Masayuki Koshikawa, Takehiro Ito, Eiichi Watanabe, Yukio Ozaki, Hideo Izawa

**Affiliations:** 1 Department of Cardiology, Fujita Health University, 1-98 Dengakugakubo, Kutsukake-Cho, Toyoake, Aichi 4701192 Japan; 2 Information Technology and Management Center, Shiga University of Medical Science, Seta Tsukinowa-Cho, Otsu, Shiga 5202192 Japan

**Keywords:** Atrial fibrillation, Catheter ablation, Rotor, Phase mapping, Pulmonary vein isolation

## Abstract

Persistent atrial fibrillation (PeAF) may develop arrhythmogenic substrates of rotors/multiple wavelets. However, the ways in which pulmonary vein isolation (PVI) affects the dynamics of rotor/multiple wavelets in PeAF patients remain elusive. Real-time phase-mapping (ExTRa mapping, EXT) in the whole left atrium (LA) was performed during PeAF before and after PVI (*n* = 111). The percentage of time in which rotor/multiple wavelets (phase singularities) was observed during each 5-s phase-mapping recording (non-passive activation ratio, %NP) was measured as an index of its burden. The mapping areas showing %NP ≥ 50% were defined as rotor/multiple-wavelet substrates (RSs). Before PVI, RSs were globally distributed in the LA. After PVI, %NP decreased (< 50%) in many RSs (PVI-modifiable RSs) but remained high (≥ 50%) in some RSs, especially localized in the anterior/septum/inferior regions (PVI-unmodifiable RSs, 2.3 ± 1.0 areas/patient). Before PVI, vagal response (VR) to high-frequency stimulation was observed in 23% of RSs, especially localized in the inferior region. VR disappearance after PVI was more frequently observed in PVI-modifiable RSs (79%) than in PVI-unmodifiable RSs (55%, *p* < 0.05), suggesting that PVI affects autonomic nerve activities and rotor/multiple wavelet dynamics. PVI-unmodifiable RSs were adjunctively ablated in 104 patients. The 1-year AT/AF-free survival rate was 70% in those with PVI alone (*n* = 115), and 86% in patients with the adjunctive ablation (log-rank test = 7.65, *p* < 0.01). PVI suppresses not only ectopic firing but also rotor/multiple wavelets partly via modification of autonomic nerve activities. The adjunctive ablation of PVI-unmodifiable RSs improved the outcome in PeAF patients and might be a novel ablation strategy beyond PVI.

**Supplementary Information:**

The online version contains supplementary material available at 10.1007/s00380-022-02209-6.

## Introduction

Pulmonary vein isolation (PVI) is the cornerstone of catheter ablation for atrial fibrillation (AF) but is associated with limited success rates in patients with persistent AF (PeAF) [1–3]. However, there is no consensus on the additional ablation strategy beyond PVI in PeAF.

Optical mapping experiments have demonstrated that single rotor and/or multiple wavelet excitations play a role in AF maintenance [4, 5]; their elimination and/or modification are proposed as an effective ablation strategy for PeAF [6]. A previous non-invasive mapping study reported that PeAF was maintained predominantly by drivers clustered in a few regions. Most of them were single meandering or multiple reentrant activities recurring in the same region; these can be targeted as the arrhythmogenic substrate in PeAF ablation [7]. However, in humans, the mapping technique and the imaging quality have been limited to visualizing the rotor/multiple wavelet excitations, and thus the impact of PVI on the complex reentrant activities in PeAF also remains elusive.

ExTRa Mapping™ (EXT), a custom-made mapping system (Nihon Kohden, Tokyo, Japan), can create phase maps (PMs) in real time by combining actual atrial electrograms and computerized virtual action potentials in human AF. EXT can automatically calculate the percentage of time in which single/multiple rotational excitations are observed within the recording area (non-passive activation ratio, %NP) to quantify the burden of rotor/multiple wavelet excitations [8–10]. In an experimental study, PMs provided by EXT are consistent with those created by high-resolution optical mapping [10]. EXT can provide images that were clear enough to analyze the reentry dynamics in human AF-like optical mapping system.

The aims of this study are to examine the impact of PVI on the rotor/multiple wavelet dynamics in PeAF using EXT and to seek an adjunctive ablation strategy beyond PVI.

## Materials and methods

### Patients

**Fig. 1:**
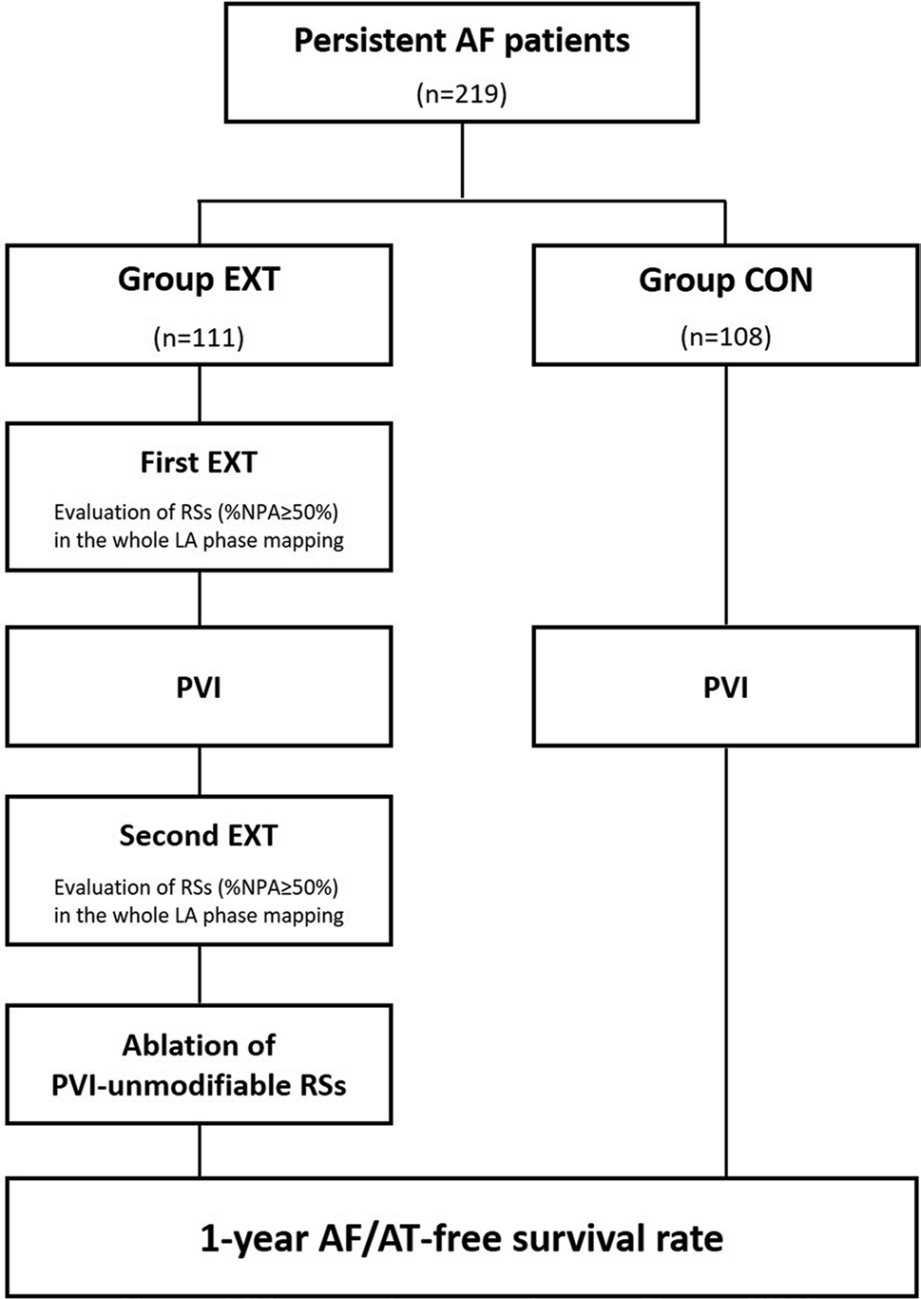
Study protocol

Two hundred nineteen consecutive PeAF patients undergoing index catheter ablation at the Fujita Health University from 2017 November to 2021 January were eligible (Fig. 1). The protocol was approved by the review board of Fujita Health University School of Medicine and written informed consent was obtained from all patients (HM20-273). The study was conducted in accordance with the ethical standards of the Declaration of Helsinki. Baseline demographics and clinical information were obtained, and laboratory examinations were performed before the procedure. Transthoracic echocardiography was performed before catheter ablation to assess left-atrial diameter (LAD), left-ventricular (LV) systolic/diastolic dimensions, and left-ventricular ejection fraction (LVEF). A 3-dimensional image of the left-atrial (LA)/pulmonary vein geometry was reconstructed by cardiac computed tomography imaging.

All patients received oral anticoagulation therapy with vitamin K antagonist (VKA, international normalized ratio: 2.0–3.0) or non-vitamin K antagonist oral anticoagulant (NOAC) for ≥ 4 weeks prior to the catheter ablation. The anticoagulants were used without interruption during the procedure. Transesophageal echocardiography was performed 1 day before catheter ablation to detect LA thrombus. All antiarrhythmic drugs (AAD) were stopped 5 days prior to the procedure.

Exclusion criteria are as follows: patients with previously diagnosed structural heart disease, cardiac sarcoidosis, and mechanical valves; patients under 18 years old and those who were pregnant; patients with creatinine clearance (calculated by Cockcroft-Gault formula) < 15 mL/min and those on hemodialysis; and patients who had LA appendage thrombus on transesophageal echocardiography before the procedure.

PeAF patients were assigned to two groups before the procedure based on physician’s choice/preference: Group EXT (*n* = 111) was to receive PVI with EXT-based real-time phase mapping (RT-PM) and adjunctive substrate ablation; Group CON (*n* = 108) was to receive only PVI without RT-PM and adjunctive ablation (Fig. 1). The ablation outcomes were prospectively evaluated during the follow-up period.

### Real-time phase mapping

**Fig. 2:**
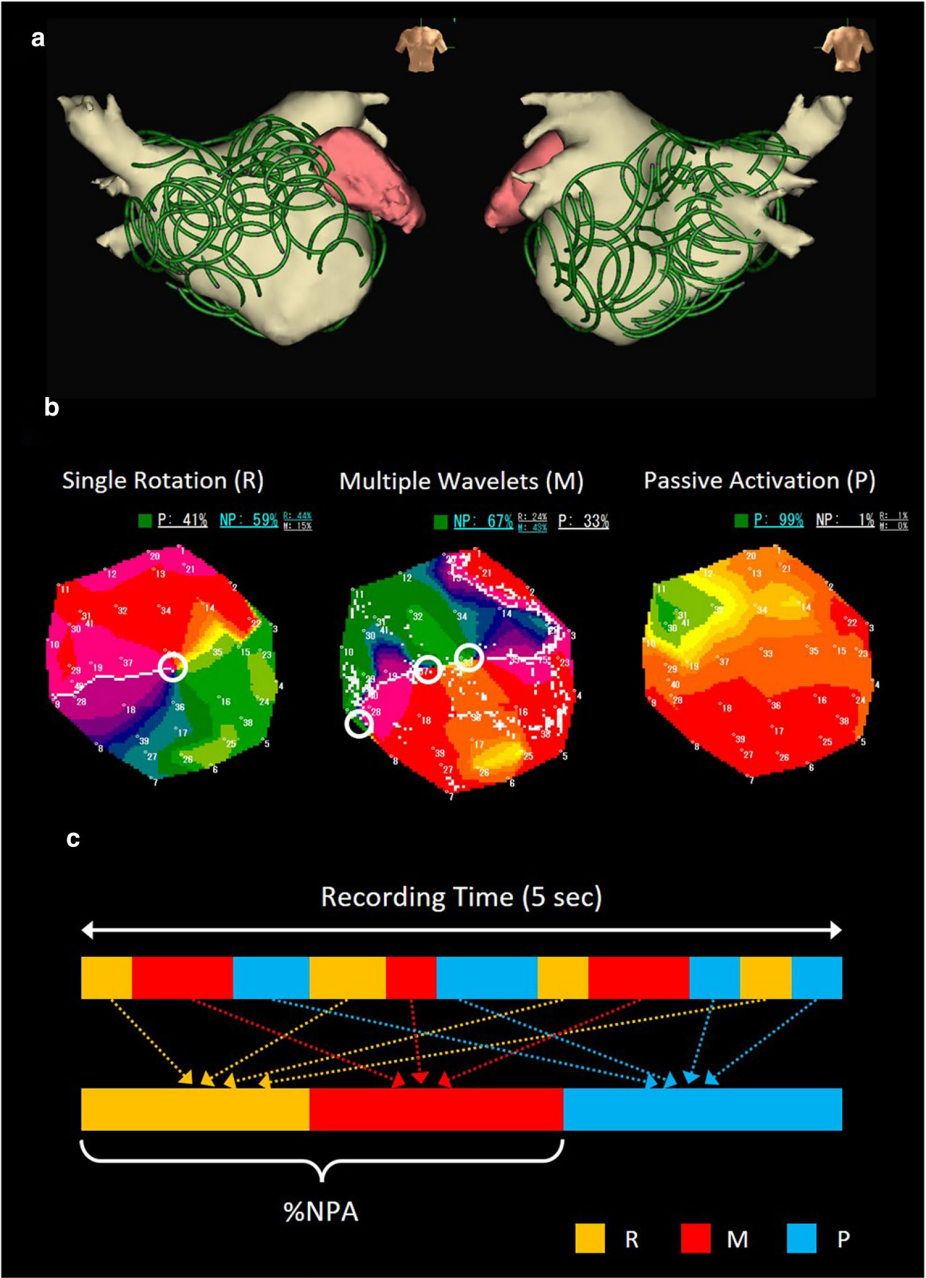
Excitation patterns of reentrant activities. **a**, Whole LA mapping with a 20 polar spiral-shaped mapping catheter. **b**, Excitation patterns of reentrant activities in real-time phase mapping. Representative phase maps (PMs) were shown. Open white circle indicates phase singularity (PS). Single rotation (R, left): PS = 1. Multiple wavelets (M, middle): PS ≥ 2. Passive activation (P, right): PS = 0. **c**, Non-passive activation ratio (%NP). %NP: time ratio of non-passive activation (*R* + *M*) to 5-s recording

RT-PM was performed using EXT in the whole LA before PVI. EXT was based on 41 bipolar intra-atrial electrograms (including 9 virtual electrograms) recorded by a deflectable 20-pole spiral-shaped catheter with a diameter of 2.5 cm (Reflexion HD™, Abbott, Fig. 2a). The contact was confirmed by the recorded electrograms, fluoroscopy, and 3-dimensional mapping geometry. The distance between a mapping point and the geometry surface created by EnSite NavX was set at 5 mm. The data sampling was adopted as good contact at the areas where sufficient electrograms could be recorded from most of the electrodes. When sufficient electrograms were not detected, the sensing threshold was decreased from 0.03 mV to 0.01 mV. Based on the 5-s recording of wave dynamics, EXT created PMs in real time via combining actual atrial electrograms and computerized virtual action potentials. EXT evaluated the excitation patterns of reentrant activities (phase singularities, PSs): a single rotational excitation (*R*, PS = 1), multiple wavelets (*M*, PSs ≥ 2), and passive activation (*P*, PS = 0, Fig. 2b). The value of the “non-passive activation ratio” (%NP), the percentage of time in which rotational activities were observed during 5-s recording, was automatically calculated in each mapping area to quantify the burden of the reentrant activities (Fig. 2c) [8–11]. The time-ratio of *M* (%*M*), *R* (%*R*), and *M* to *R* (*M*/*R*, an index of complex and disorganized reentrant activities) during the non-passive activation were also calculated. Repetitive RT-PM in the same area and in an area overlapping (> 50%) with a neighboring one confirmed the reproducibility of the excitation dynamics.

When EXT showed %NP ≥ 50%, the mapping area was defined as a “rotor/multiple wavelet substrate” (RS) with high burden of the complex reentrant activities. The whole LA was divided into the 6 regions: anterior, roof, posterior, inferior, lateral, and septal walls. The anatomical distribution of the RSs was also evaluated.

RT-PM was repeated after PVI unless sinus rhythm was restored during the procedure. The RSs detected before PVI were remapped using EXT. When the RSs still showed %NP ≥ 50% after PVI, they were defined as “PVI-unmodifiable RSs.”

### PVI

Ablation procedure was performed under deep sedation as previously reported [12]. Esophageal temperature was monitored throughout the procedure; temperature limit was set to 41 ^°^C. The procedure was performed during AF. Only radiofrequency ablation was used for PVI. No additional linear ablation in the LA was performed; only cavotricuspid isthmus ablation was permitted for documented typical atrial flutter.

PVI was achieved using a focal “point-by-point” catheter approach, delivering radiofrequency energy to the cardiac tissue with irrigation tip catheters (THERMOCOOL SMARTTOUCH® SF™, Biosense Webster, Diamond Bar, CA, USA [target contact force: 10-20 g, RF time: 30–60 s, irrigation flow rate: 8 ml/min for ≤ 30 W, 15 ml/min for > 30 W, power control mode] or FlexAbility™, Abbott, St. Paul, MN, USA [RF time: 30–60 s, irrigation flow rate: 10 ml/min for < 38 ^°^C, 13 ml/min for ≥ 38 ^°^C, temperature control mode]). RFCA lesion sets encircled the PV antra using electro-anatomical mapping (CARTO3, Biosense Webster, Diamond Bar, CA, USA or EnSite NavX, Abbott, St. Paul, MN, USA) and fluoroscopy guidance.

Activated clotting time (ACT) was measured every 20 min after the first heparin shot and additional heparin boluses were given to maintain the ACT ≥ 300 s.

### High frequency stimulation-evoked vagal response

In group EXT, high-frequency stimulation (HFS) was applied to the RSs before PVI to evaluate the association between vagal response (VR) and rotor/multiple wavelet dynamics. HFS was delivered using a bipolar ablation catheter (cycle length: 50 Hz, pulse width: 1.0 ms, and output voltage: 20 V). HFS was not applied to the RSs close to the mitral annulus which show ventricular far-field potential or ventricular capture during high-output (20 V) atrial pacing (100 beat/min) to avoid HFS-induced ventricular fibrillation. A positive VR was defined as 50% increase in the *R*–*R* interval. After PVI, HFS was repeated in the same RSs showing a positive VR before PVI and VR was reevaluated.

### Adjunctive RS ablation

In group EXT, PVI-unmodifiable RSs were adjunctively ablated. The adjunctive ablation was performed with point-by-point technique (30–35 W for 30–60 s per ablation point) with irrigation tip catheters (FlexAbility™, Abbott, St. Paul, MN, USA [RF time: 30–60 s, irrigation flow rate: 10 ml/min for < 38 °C, 13 ml/min for ≥ 38 ^°^C, temperature control mode]) to create a coin lesion (≤ 3.0–4.0 cm^2^) within each area of the PVI-unmodifiable RSs. When a coin lesion was close (< 1 cm) to a neighboring one and/or to a PVI lesion, the two were connected by RF application. After the adjunctive ablation, RT-PM was repeated around each coin lesion to re-evaluate the %NP; when an area still showed %NP ≥ 50% after PVI, the additional adjunctive ablation was performed in that area. End point of the adjunctive ablation was disappearance of all PVI-unmodifiable RSs (%NP < 50%).

When AF was sustained after PVI and the adjunctive ablation, internal (3–35 J) or external (50–220 J) electrical cardioversion was performed to restore sinus rhythm.

### Confirmation of residual potentials and/or conduction gaps in PVI

After restoration of sinus rhythm, electro-anatomical mapping was performed. If there were residual PV potentials and/or conduction gaps within the isolated PV, additional RF ablation was performed to eradicate them. Conduction block from the PV to the LA was confirmed by high-output pacing (20 V output, 1 ms pulse) from the isolated PV area. After a 20-min waiting period, boluses of isoproterenol (2–4 μg) and adenosine triphosphate (20 mg) were administered. If spontaneous PV reconnection and/or drug-evoked dormant conduction were observed, additional RF was applied to eliminate it.

### Follow-up

All patients were followed up by cardiologists at the outpatient department in Fujita Health University at 1, 3, 6, 9, and 12 months after ablation. The outcomes were prospectively evaluated. All patients were asked about their symptoms and underwent a 12-lead electrocardiogram in each follow-up. At the 6- and 12-month follow-ups, 24-h Holter ECG monitoring was performed. If atrial tachycardia (AT) and/or AF recurred within a 3-month blanking period, AAD (bepridil or amiodarone) was prescribed and then discontinued after the blanking period. However, the continuation of AAD after the blanking period was allowed at the cardiologists’ discretion. AT/AF recurrence was defined as any atrial tachy-arrhythmias lasting more than 30 s which occurred after the blanking period. The 1-year AT/AF-free survival rates were evaluated in Group EXT and Group CON.

### Statistical analysis

Continuous variables, represented as mean ± standard deviation, were compared using unpaired t-tests. Categorical data, expressed as frequencies and percentages, were compared using chi-square tests. The follow-up period was calculated from the date of catheter ablation to that of the AF recurrence or censoring. AT/AF-free survival rate was calculated using Kaplan–Meier survival analysis, and log-rank statistics were used for group comparisons.

All tests were 2-sided, and a *p*-value < 0.05 was considered statistically significant. Statistical analyses were performed using JMP14 (SAS Institute, Cary, NC, USA).

## Results

### Patient characteristics

Table 1 shows the baseline patient characteristics. Average age was 66 ± 9 years old. Mean CHA_2_DS_2_-VASc scores were 2.5 ± 1.6 points. In echocardiography, LAD was 42.4 ± 5.6 mm and LVEF was 52.9 ± 10.0%. The plasma levels of NT-ProBNP were 864 ± 657 pg/ml. The percentage of long PeAF (> 1 year) was 31%. There was no significant difference in baseline characteristics between Group EXT and Group CON (Table 1).

**Table 1:** Patient characteristics

	All patients (*n* = 219)	Group EXT (*n* = 111)	Group CON (*n* = 108)	*P*-value
Age, y/o	66 ± 9	65 ± 10	67 ± 9	0.1539
Female, *n* (%)	52 (24)	28 (25)	24 (22)	0.6014
Body mass index, kg/m^2^	24.7 ± 3.8	24.8 ± 4.1	24.5 ± 3.4	0.6840
LS PeAF, *n* (%)	68 (31)	37 (33)	31 (29)	0.4589
CHADS_2_ sore, points	1.6 ± 1.2	1.5 ± 1.3	1.7 ± 1.3	0.3377
CHA_2_DS_2_-VASc sore, points	2.5 ± 1.6	2.3 ± 1.6	2.7 ± 1.6	0.0859
Congestive heart failure, *n* (%)	104 (47)	50 (45)	54 (50)	0.4628
Hypertension, *n* (%)	130 (59)	63 (57)	67 (62)	0.4262
Age ≥ 75, *n* (%)	40 (18)	22 (20)	18 (17)	0.5457
Diabetes, *n* (%)	41 (19)	22 (20)	19 (18)	0.6726
Stroke/TIA, *n* (%)	17 (8)	7 (6)	10 (9)	0.4133
Laboratory Data				
NT-proBNP, pg/ml	864 ± 657	826 ± 611	903 ± 702	0.3886
BUN, mg/dl	14.7 ± 4.7	14.2 ± 4.7	15.3 ± 4.7	0.0971
Creatinine, mg/dl	0.86 ± 0.24	0.84 ± 0.20	0.89 ± 0.26	0.1056
Echocardiography				
LVEF, %	52.9 ± 10.0	53.7 ± 9.4	52.3 ± 10.4	0.2810
LVDd, mm	47.7 ± 6.5	47.7 ± 6.1	47.8 ± 6.9	0.8826
LVDs, mm	34.5 ± 7.6	33.9 ± 6.7	35.1 ± 8.4	0.2094
LAD, mm	42.4 ± 5.6	42.4 ± 5.2	42.4 ± 6.0	0.9987
LAVI, ml/m^2^	45.2 ± 12.0	45.0 ± 11.4	45.3 ± 12.7	0.8044
Medication				
NOAC, *n* (%)	204 (93)	104 (94)	100 (93)	0.7470
ACEI/ARB, *n* (%)	109 (49)	53 (48)	56 (52)	0.5436
β-blocker, *n* (%)	119 (54)	55 (49)	64 (59)	0.1489

*ACEI/ARB* angiotensin converting enzyme inhibitor/angiotensin 2 receptor blocker, *LAD* left-atrial diameter, *LAVI* left-atrial volume index, *LVEF* left-ventricular ejection fraction, *LVDs/d* left-ventricular systolic/diastolic dimension, *LS PeAF* long-standing persistent atrial fibrillation, *NOAC* non-vitamin K dependent oral anticoagulant, *NT-proBNP* N-terminal pro-brain natriuretic peptide, *TIA* Transient ischemic attack

### Real-time phase mapping before PVI

**Fig. 3:**
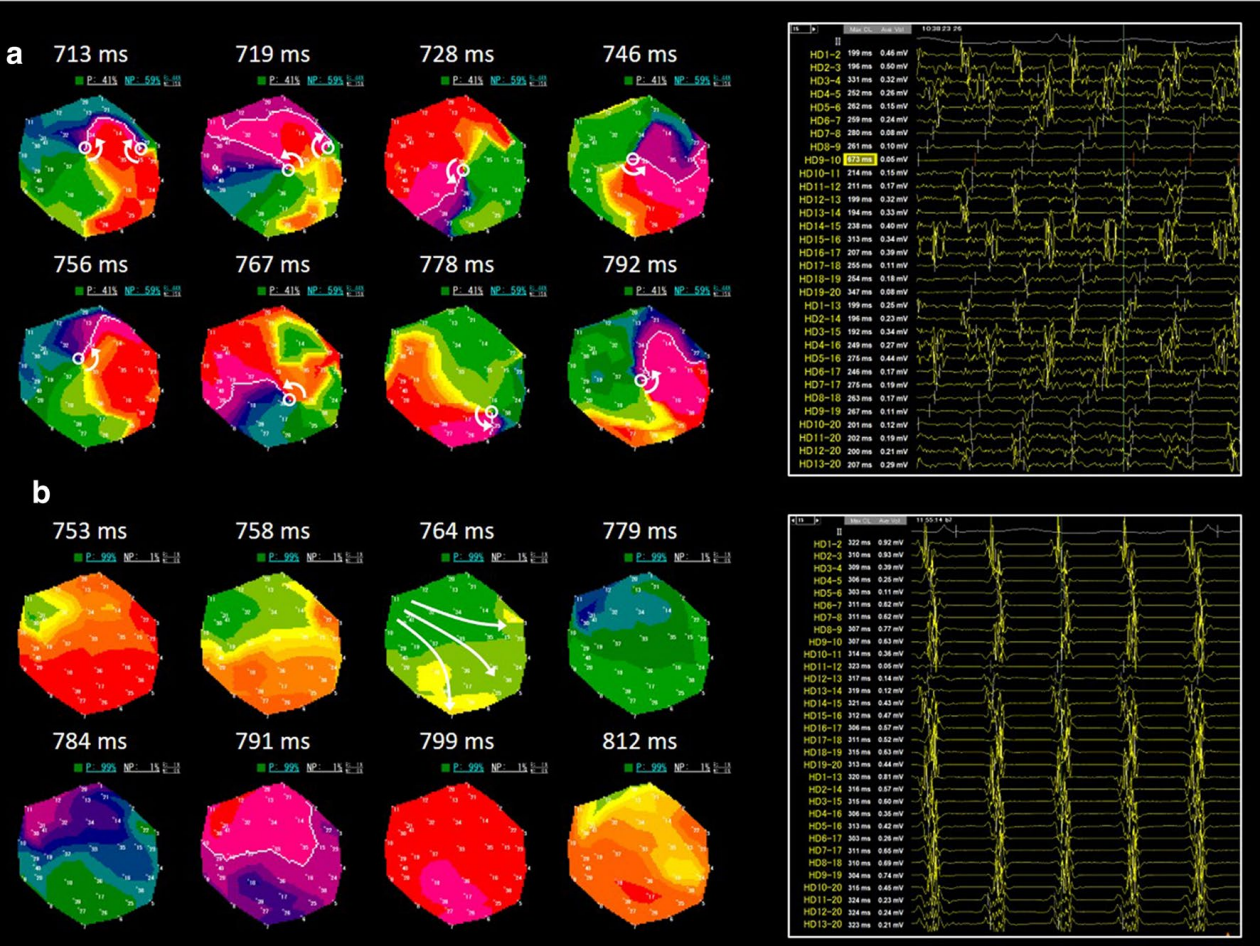
Real-time phase mapping in EXT. **a**, Representative PMs (left) and intracardiac electrocardiogram (IEGM, right) in high %NP (≥ 50%) area. **b**, PMs (left) and IEGM (right) in low %NP (< 50%) area. IEGM was recorded using a 20 polar spiral-shaped mapping catheter. Open white circle indicates phase singularity

In Group EXT (*n* = 111), RT-PM was performed using EXT and the PMs covering the whole LA were created before PVI (Fig. 2a). The average time for RT-PM was 12 ± 3 min and the average number of PMs acquired for the whole LA mapping was 27 ± 5. Figure 3a shows representative PMs and simultaneous recording of intracardiac electrograms (IEGM) in an RS (%NP ≥ 50%). In this area, the %NP was 59%, meaning that PSs were observed for 3.0 s per 5.0-s recording. In the PMs, a single meandering rotor (PS = 1) and multiple wavelets (PSs ≥ 2) were frequently observed and dynamically interchanged; the percentage of time of *R* (%*R*) and *M* (%*M*) to a 5.0-s recording were 44% and 15%, respectively, and the time-ratio of *M* to *R* (*M*/*R*, an index of disorganized excitations) was 0.34 (Fig. 3a, Supplemental Movie 1). No stable rotor was observed. The generation/extinction of PSs due to wave breakup/collision was frequently observed during the recording period. The complex spatiotemporal behavior of rotor/multiple wavelets was repeatedly observed in the RS. The IEGM exhibited the high-frequency and periodic excitation patterns, being similar to the spatiotemporal dispersion electrogram (SDE) associated with AF drivers reported by Seitz et al. (Fig. 3a) [13]. Figure 3b shows representative PMs and IEGM in a low %NP (< 50%) area. In this area, %NP was 1% and almost no PS was observed. The IEGM exhibits organized excitations, representing the passive wave propagation (Fig. 3b, Supplemental Movie 2).

**Fig.4:**
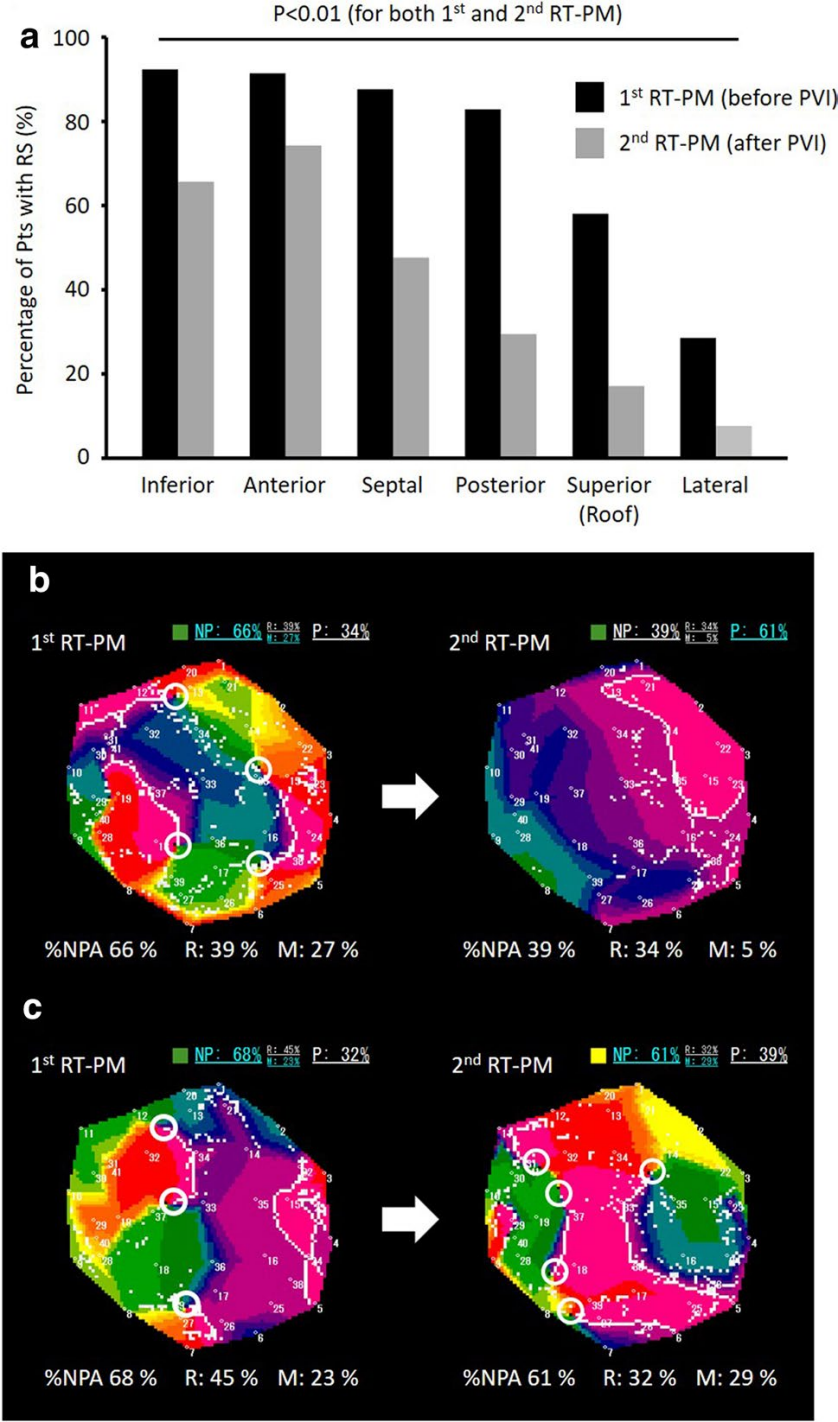
Distribution of rotor/multiple wavelet substrates (RSs). **a**, Distribution of RSs (%NP ≥ 50%) in real-time phase mapping before and after PVI. **b**, Representative PMs of a PVI-modifiable RS. **c**, Representative PMs of a PVI-unmodifiable RS

In the first RT-PM, 612 RSs were detected and analyzed: average %NP was 62.3 ± 9.8% (Table 2). Figure 4a shows the anatomical distribution of RSs in the RT-PM before PVI. Among the 6 regions, the RSs were frequently observed in the anterior, inferior, septal, and posterior regions.

**Table 2:** Excitation dynamics in PVI-modifiable and PVI-unmodifiable RSs

	Total RS (*n*=612)			PVI-M RS (*n*=345)			PVI-UM RS (*n*=267)			*P* value (PVI-M RS vs. PVI-UM RS)	
	1st EXT	2nd EXT	*P* value	1st EXT	2nd EXT	*P* value	1st EXT	2nd EXT	*P* value	1st EXT	2nd EXT
%NPA, %	62.3±9.8	52.5±18.4	<0.001	61.4±9.4	32.0±14.2	<0.001	63.1±10.2	62.3±10.4	0.1991	0.0055	<0.001
%*R*, %	37.2±8.3	32.8±12.4	<0.001	37.1±8.4	21.5±9.8	<0.001	37.2±8.3	38.2±9.4	0.006	0.7533	<0.001
%*M*, %	25.1±8.8	19.6±10.5	<0.001	24.2±8.8	10.7±6.6	<0.001	25.9±8.7	23.9±9.2	<0.001	0.0020	<0.001
*M*/*R*	0.72±0.35	0.62±0.35	<0.001	0.70±0.34	0.51±0.34	<0.001	0.75±0.35	0.68±0.36	<0.001	0.0509	<0.001

*EXT* extra mapping, *PVI-M RS* PVI-modifiable reentry substrate, *PVI-UM RS* PVI-unmodifiable reentry substrate, *RS* reentry substrate, *%M* percentage of multiple wavelets (multiple phase singularities), *%S* percentage of single rotation (single phase singularity)

### PVI

PVI was successfully performed in all patients. AF was terminated during PVI in 13 patients (7 in Group EXT and 6 in Group CON).

### Real-time phase mapping after PVI

The second RT-PM was performed after PVI in 104 patients of Group EXT; it could not be performed during sinus rhythm in 7 patients who had AF termination by PVI. The RSs detected in the first RT-PM significantly decreased %NP, %R, %M, and M/R in the second RT-PM, suggesting that the disorganized excitation dynamics became more organized after PVI (Table 2). Figure 4a shows the anatomical distribution of the RSs in the second RT-PM. Many RSs, detected especially in the roof, posterior, and lateral regions in the first RT-PM, decreased the %NP in the second RT-PM, suggesting that PVI can, at least partly, modify the excitation dynamics of RSs even in the area away from the PVI lesions. Among 612 RSs detected in the first RT-PM, %NP significantly decreased (< 50%) in 345 RSs (PVI-modifiable RSs) in the second RT-PM but remained high (≥ 50%) in 267 RSs (PVI-unmodifiable RSs, Table 2). Representative snapshots of PMs in PVI-modifiable and PVI-unmodifiable RSs are shown in Fig. 4b, c.

### Vagal response to high-frequency stimulation

**Fig. 5:**
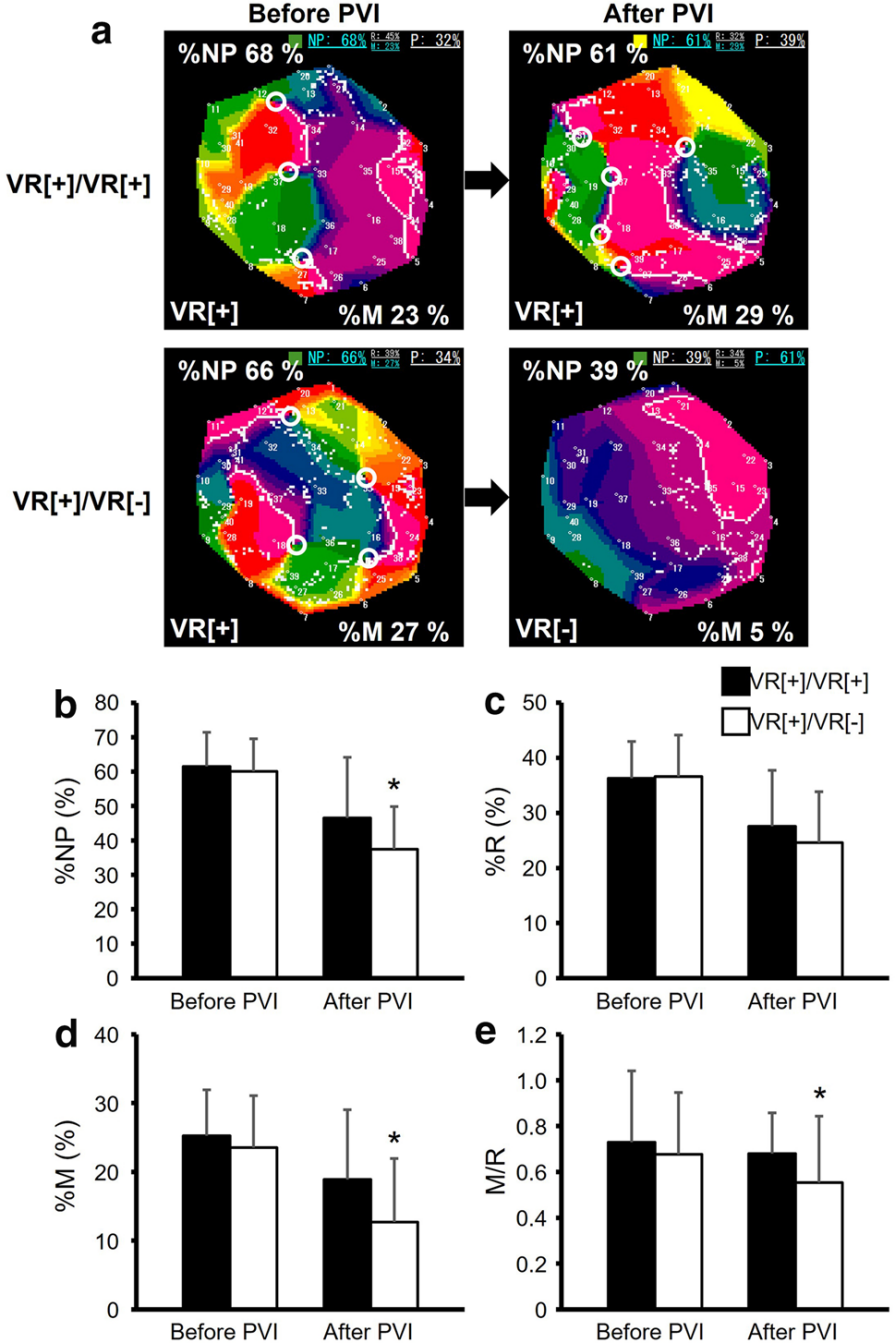
Vagal response (VR) to high frequency stimulation (HFS) in RSs. **a**, Representative PMs of VR[ +]/VR[ +] and VR[ +]/VR[ – ]. **b–e**, %NP, %*R*, %*M*, and *M*/*R* before and after PVI. Black box: RSs which showed positive VRs before and after PVI (VR[ +]/VR[ +], *n* = 30). White box: RSs which showed a positive VR before PVI but did a negative VR after PVI (VR[ +]/VR[ – ], *n* = 80)

HFS were applied to the RSs (*n* = 505) before PVI; 110 RSs had a positive VR (VR[ +]) but 395 did not (VR[-]). The RSs with VR[ +] were distributed mainly in the LA bottom, away from PVI lesions (bottom: 48%, posterior: 24%, anterior: 17%, roof: 6%, septum: 5%). The values of %NP, %*R*, %*M*, and *M*/*R* were not statistically different between the RSs with VR[ +] and VR[-] (VR[ +] vs. VR[-]: 61 ± 9% vs. 62 ± 9% for %NP, 37 ± 7% vs. 37 ± 8% for %*R*, 25 ± 7% vs. 26 ± 13% for %M, and 0.71 ± 0.28 vs. 0.77 ± 0.48 for M/R). After PVI, HFS was repeated in the same areas of RSs showing VR[ +] before PVI; the 80 RSs (73%) changed the HFS response from VR[ +] to VR[-] (VR[ +]/VR[-]) whereas the 30 RSs did not (VR[ +]/VR[ +]). Representative PMs in VR[ +]/VR[ +] and VR[ +]/VR[ – ] are shown in Fig. 5a. In the first RT-PM, %NP, %*R*, %*M*, and *M*/*R* were unchanged between VR[ +]/VR[ – ] and VR[ +]/VR[ +] (VR[ +]/VR[ – ] vs. VR[ +]/VR[ +]: 60 ± 9% vs. 62 ± 10% for %NP, 37 ± 6% vs. 36 ± 7% for %*R*, 24 ± 7% vs. 25 ± 9% for %M, and 0.68 ± 0.27 vs. 0.73 ± 0.31 for *M*/*R*, Fig. 5b, c, d, e). However, in the second RT-PM, %NP, %M, and *M*/*R* were significantly lower in the areas with VR[ +]/VR[-] compared to those with VR[ +]/VR[ +] (VR[ +]/VR[-] vs. VR[ +]/VR[ +]: 37 ± 12% vs. 47 ± 18% for %NP, 13 ± 6% vs. 19 ± 8% for %M, and 0.55 ± 0.29 vs. 0.68 ± 0.18 for *M*/*R*, *p* < 0.05, Fig. 5b, d, e) although %R was unchanged (25 ± 9% vs. 28 ± 10%, Fig. 5c). VR[ +]/VR[ – ] showed significantly greater reduction of %NP and %*M* from the first to the second RT-PM than VR[ +]/VR[ +] (VR[ +]/VR[ – ] vs. VR[ +]/VR[ +]: 23 ± 14% vs. 15 ± 18% for Δ%NP and 11 ± 9% vs. 6 ± 11% for Δ%*M*, *p* < 0.05). VR[ +]/VR[ – ] also showed greater decrease in M/R than VR[ +]/VR[ +] but the difference did not reach statistical significance (0.15 ± 0.42 vs. 0.05 ± 0.35 for Δ*M*/*R*).

Among the 110 RSs with a positive VR before PVI, 81 were classified as PVI-modifiable RSs after PVI and 29 were classified as PVI-unmodifiable RS: VR disappearance after PVI was more frequently observed in PVI-modifiable RSs (79%, 64/81) than in PVI-unmodifiable RSs (55%, 16/29, *p* < 0.05).

These suggests that the disorganized reentrant excitation became organized ones associated with VR-change after PVI; autonomic nerve activity and rotors/multiple wavelet dynamics can be, at least partly, modified by PVI.

### PVI-unmodifiable RS ablation

**Fig. 6:**
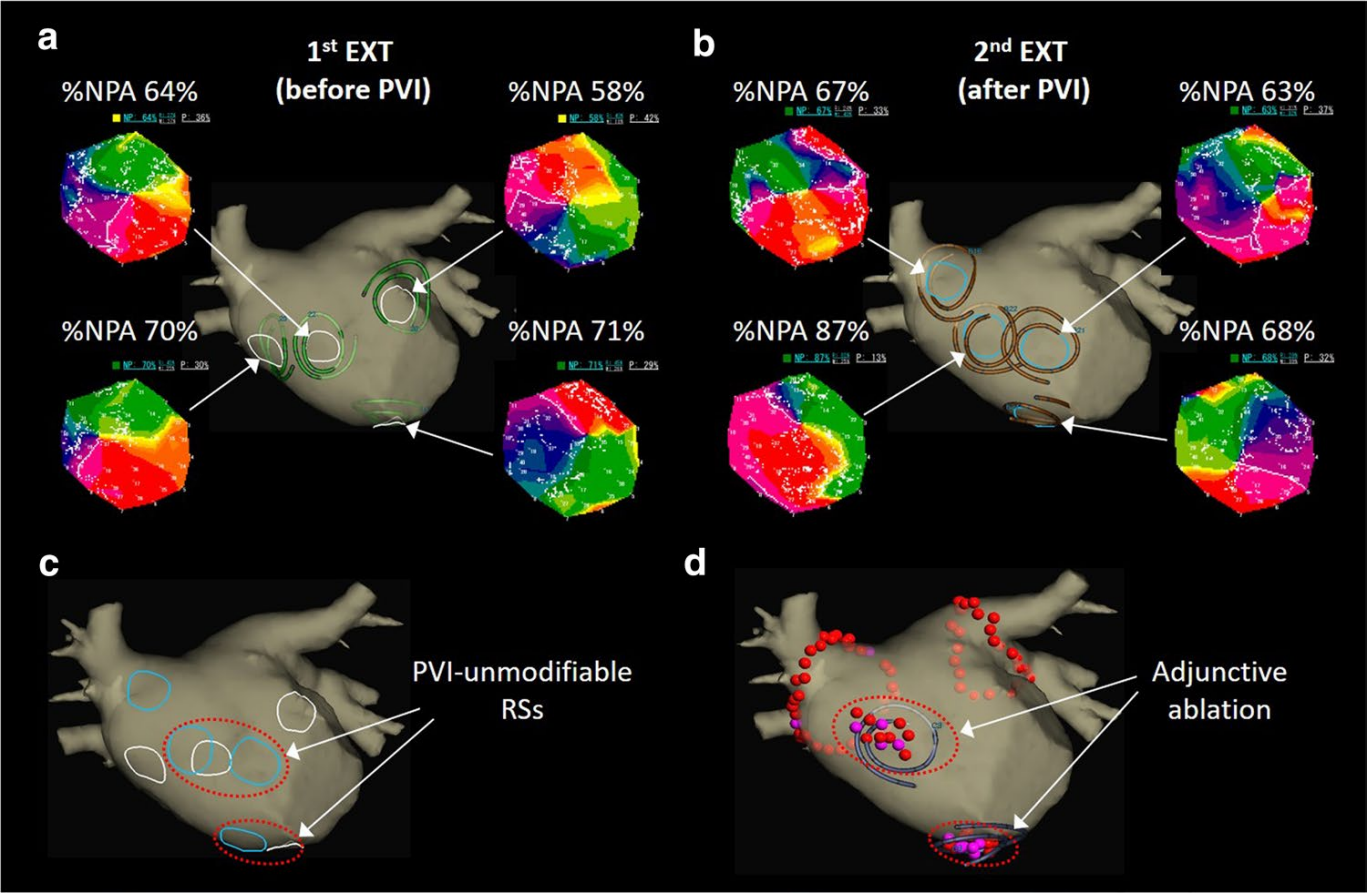
Adjunctive ablation targeting PVI-unmodifiable RSs. **a**, RS distribution in real-time phase mapping before PVI. **b**, RS distribution after PVI. **c**, PVI-unmodifiable RSs. **d**, Adjunctive ablation targeting PVI-unmodifiable RSs

In Group EXT, the adjunctive ablation targeting the PVI-unmodifiable RSs was performed with point-by-point technique with irrigation tip catheters to create a coin lesion within each area of the PVI-unmodifiable RSs (Fig. 6). The 7 patients in Group EXT received only PVI since the second RT-PM could not be performed during sinus rhythm and the adjunctive ablation was not required after PVI.

The average number of adjunctive ablation lesions was 2.3 ± 1.0 per patient and the average area of the adjunctive ablation was 2.6 ± 0.8 cm^2^ per lesion. AF was terminated in 2 patients during the ablation. After PVI-unmodifiable RS ablation, the third RT-PM was performed around each lesion to confirm a decrease in %NP (< 50%). Finally, AF was terminated by internal or external cardioversion in all patients.

### Complications and follow-up

**Fig. 7:**
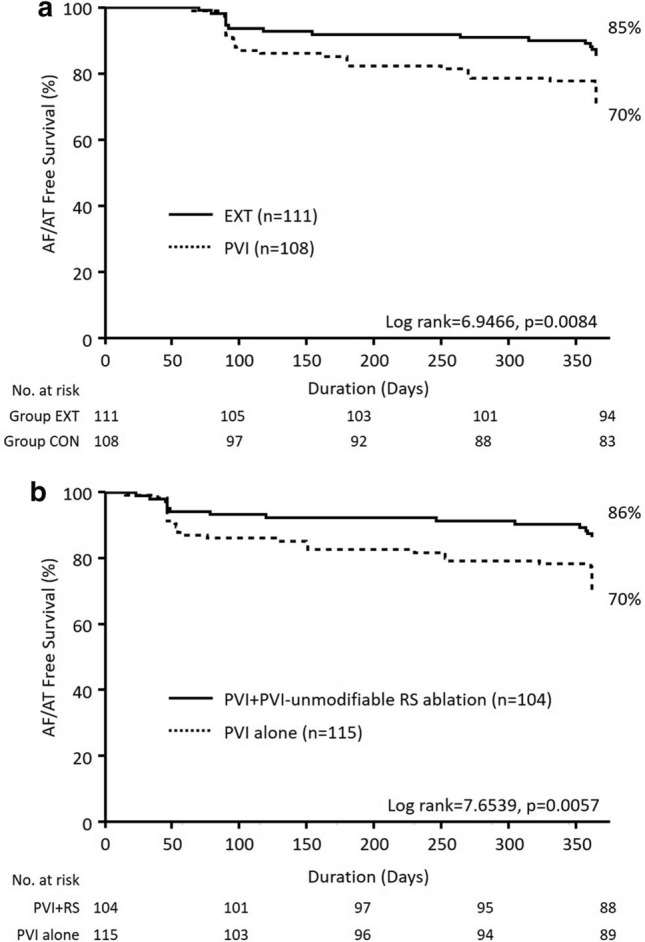
1-year AF/AT Free survival rate. **a**, 1-year atrial tachycardia (AT)/AF free survival rate in Group EXT (*n* = 111) and Group CON (*n* = 108). **b**, 1-year AT/AF free survival rate in PVI + adjunctive ablation (*n* = 104) and PVI alone (*n* = 115)

No major procedure-related complications occurred in either Group EXT or Group CON during or after the procedure. AAD was prescribed in 20 patients after the procedure (Group EXT: *n* = 11, Group CON: *n* = 9, *p* = 0.73). Forty-eight patients had AF recurrence after the 3-month blanking period (*n* = 16 in Group EXT, *n* = 32 in Group CON). Among them, 4 patients in Group EXT and 8 patients in Group CON had AT recurrence (*p* = 1.00). Kaplan–Meier analysis demonstrated that Group EXT had a significantly higher 1-year AF/AT-free survival rate than Group CON (85% vs. 70%, log-rank = 6.69, *p* < 0.01, Fig. 7a). The 7 patients in Group EXT did not receive the adjunctive ablation since sinus rhythm was restored after PVI. Therefore, the 1-year AF/AT-free survival rate was also compared between patients with and without the adjunctive ablation after PVI. Patients with PVI-unmodifiable RS ablation (*n* = 104) after PVI also had a significantly higher 1-year AF/AT-free survival rate than those with PVI alone (*n* = 115, 86% vs. 70%, log-rank = 7.65, *p* < 0.01, Fig. 7b).

## Discussion

The major findings of this study are as follows. RSs were distributed mainly in the anterior, inferior, septal and posterior regions in PeAF patients; there was a region hierarchy of the burden of rotor/multiple wavelet excitations. In addition, PVI could at least partly modify the excitation dynamics of RSs, associated with the modification of autonomic nerve activities; however, there still remain areas showing high %NP (≥ 50%, PVI-unmodifiable RSs), especially in the anterior and inferior regions. A third finding is that PVI with adjunctive ablation targeting the PVI-unmodifiable RSs had a higher 1-year AF/AT-free survival rate than PVI alone. EXT is effective in analyzing the complex and repetitive reentrant activities during PeAF and in identifying the critical target sites for the adjunctive ablation based on rotor/multiple wavelet dynamics.

### Comparison with previous studies

The multiplication of randomly circulating waves associated with electrical and structural atrial remodeling underlies the maintenance of PeAF. PVI is a cornerstone for paroxysmal AF ablation but is not sufficient for PeAF ablation; various potential substrates of rotors/multiple wavelets in PeAF have been empirically ablated. Nademanee et al. ablated the areas showing complex fractionated atrial electrogram (CFAE), associated with random reentry and fibrillatory conduction. In most cases, adjunctive CFAE ablation could terminate PeAF during the procedure [14]. However, a randomized clinical trial failed to show the efficacy of CFAE ablation; the extensive adjunctive ablation (linear or/and CFAE ablation) beyond PVI had a similar success rate to PVI alone (around 60%) [1].

Phase-based optical mapping studies have demonstrated that spiral wave reentries with a spectrum of excitation phenomena from a single meandering rotor to multiple/periodic wavelets with a peripheral fibrillatory conduction are responsible for maintenance of cardiac fibrillation in animal models [4, 5, 15, 16]. Atrial fibrotic remodeling creates arrhythmogenic substrate causing the complex propagation and facilitating the repetitive appearance of spatially meandering rotors and multiple wavelets [17]. The ablation targeting these areas may be effective for PeAF treatment.

Narayan et al. tried to create interpolated phase movies of the atrial activation patterns and isochronal maps from individual cycles using multielectrode basket catheters. They, therefore, developed a patient-tailored approach to target focal impulse and repetitive rotational activity (rotor) which drive AF (focal impulse and rotor modulation, FIRM). They reported a high success rate of FIRM-guided ablation (82.4%) [18, 19]. However, the efficacy has not been replicated by other groups [20]. The FIRM-guided ablation is conceptually mechanistic but the potential problems are the low spatial resolution of electrode mapping (4 pixels/cm^2^) and the mismatch between basket catheter shape and atrial anatomy to detect critical arrhythmogenic substrate.

Seitz et al. ablated the areas exhibiting SDE recorded by a multipolar catheter (PentaRay) regardless of whether the electrograms are CFAE or not [13]. In optical mapping experiments with numerical simulation study, these excitation patterns represent reentrant activities that drive AF (rotors). The SDE-based ablation resulted in an 89% AF/AT-free survival rate after a single procedure in PeAF patients. In this study, the high %NP areas usually demonstrated SDE patterns and so, at least partly, coincided with the areas targeted by SDE-based ablation. Seitz et al. ablated only the areas showing SDEs without PVI, aiming for a fully patient-tailored AF intervention. At the same time, we performed PVI since we assumed that the PVs remain important in the pathophysiology of PeAF. EXT can perform RT-PM, like experimental optical mapping, and quantify the burden of rotor/multiple wavelets in real time. This may be an advantage of EXT- over SDE-based ablation. PVI plus EXT-based adjunctive ablation in our study showed a similar success rate to the value in Seitz et al.’s previous study (87%) [13].

Haissaguerre et al. performed non-invasive ECG mapping using a vest with an array of body surface 252-electrodes and a computed tomography-based cardiac geometry to detect AF drivers (CARDIO INSIGHT) [7]. PeAF was maintained predominantly by drivers clustered in a few regions, most of them being unstable reentries (80% reentries and 20% foci). The reentries were not stabilized and although they substantially meandered, they recurred in the same region, being the similar observation in our study. They also demonstrated that the reentries and focal activities colocalized, suggesting that they share a pathology and/or a functional link at cellular/tissue levels. Our study targeted only higher arrhythmogenic burden of rotational excitations but might also suppress focal breakthroughs in the vicinity. In the initial experience of CARDIO INSIGHT, 64% patients were in sinus rhythm, 22% in atrial tachycardia, and 20% in AF during 1-year follow-up. In our study, 85% of Group EXT patients were in sinus rhythm, 4% in atrial tachycardia, and 11% in AF during 1-year follow-up. The possible drawback of the non-invasive ECG mapping is lower spatial resolution, which can cause drivers to be missed, incorrectly located, or artefactually created where they do not actually exist, potentially causing excessive ablation and giving rise to the macro-reentry substrates.

Previous studies have demonstrated that the cardiac autonomic activities play a role in the initiation and maintenance of AF [21, 22]. Increased parasympathetic tones, on the one hand, activate acetylcholine-activated potassium channels, carrying repolarizing current and shortening action potential duration. On the other hand, increased sympathetic tone increases intracellular calcium load, triggering early after-depolarizations and ectopic firing [23, 24]. Baykaner et al. have demonstrated the spatial relationship between the cardiac autonomic nervous system and the region of focal impulses and rotational excitations in AF patients. The AF sources were frequently colocalized to the anatomical sites of ganglionated plexus in the left atrium. However, many AF sources were not correlated with the anatomical location of ganglionated plexus, being consistent with the findings of our study [25]. The intrinsic cardiac autonomic network is spatially interconnected by ganglionated plexus, and PVI would have an impact on the VR to HFS in the area away from the ablated lesions. Baykaner et al. ablated the AF sources without PVI. We first performed PVI and examined the impact of PVI on autonomic nerve activity and rotor/multiple wavelet dynamics; PVI per se suppressed rotor/multiple wavelets at least partly via modification of cardiac autonomic response.

### Potential advantage

Phase mapping is a common tool for optical mapping experiments to visualize the excitation patterns during cardiac fibrillation by determining the local phase of the action potential. Optical mapping in animal experiments uses photosensitive dyes to visualize cardiac action potentials, but the dyes cannot be used in humans. EXT uses a 20 polar spiral-shaped mapping catheter (Reflection HD) with the higher spatiotemporal resolution (8 pixels/cm^2^) for IEGM recording and requires full contact of the mapping catheter to the LA wall. EXT can computerize actual atrial electrograms to generate virtual action potentials in real time, and thus can create PMs like optical mapping experiments. An experimental study validated the quality and reliability of PMs created by EXT [11].

Kumagai et al. performed EXT in PeAF patients and demonstrated that PVI with posterior wall isolation suppressed the arrhythmogenic substrate with the higher burden of rotors and multiple wavelets even in the areas away from the ablated lesions [9]. The PV and its surrounding area are not only the source of AF triggers but also the site of hosting AF drivers. We performed EXT before and after PVI to detect the arrhythmogenic substrates that cannot be modified by PVI. To the best of our knowledge, this therapeutic approach has not been reported. AF-driving excitations are complex, and therefore it’s important to establish a hierarchical criterion based on activation frequency so as to discern which alleged drivers are relevant for AF maintenance [6]. EXT seems to have the advantage of quantifying the burden of the single/multiple reentrant excitations in real time.

Massive extra-PV ablation creates the substrate of iatrogenic macro-reentry. In our study, there was no difference in AT-recurrence rate between Group EXT and Group CON, indicating the amount of adjunctive extra-PV ablation may be small enough to avoid creating the iatrogenic substrates. However, the combination of PVI and PVI-unmodifiable RS ablation showed a higher 1-year arrhythmia-free survival rate than PVI alone in our study, implying that the minimized substrate ablation affects AF drivers and the outcome. The successful procedure was based on the durability of lesions ablated neither too little nor too much. The detection of individual non-PV targets provides a clinical benefit and EXT may lead to a more accurate functional classification of non-PV substrates and a more patient-tailored approach in PeAF ablation.

### Limitation

This is a single-center study with a small number of patients. The statistical power is limited and interpretations should be made with caution. Most of the patients were alternatively assigned to the two ablation strategies, PVI alone or PVI with adjunctive RS ablation, in a random manner. However, some patients with long-standing persistent AF were assigned to PVI with adjunctive RS ablation based on physician’s choice/preference when PVI alone apparently does not seem to be enough to achieve a favorable clinical outcome. This may cause selection bias. The region showing %NP ≥ 50% is defined as “RS” but there is no consensus on this definition. This study aimed at durable PVI and a decrease in %NP (< 50%) for the adjunctive RS ablation; the end point is different from that of previous studies, which does not allow comparison among techniques. The right atrium may be relevant to AF maintenance but we did not routinely perform RT-PM in the right atrium to avoid spending too much time on the index ablation.

## Conclusion

A combination of PVI and EXT-based adjunctive ablation improved the success rate of PeAF ablation. PVI-unmodifiable RSs may serve as AF drivers and play a role in the pathophysiology. EXT could visualize and evaluate the complex dynamics of rotor/multiple wavelets during AF in real time and may help us to establish a substrate-based approach for PeAF ablation.

## Supplementary Information

Below is the link to the electronic supplementary material.

Supplementary file1 (MP4 856 KB)

Supplementary file2 (MP4 456 KB)

## Data Availability

The data that support the findings of this study are available from the corresponding author upon reasonable request.
